# 1526. Time from last COVID-19 vaccination’s impact on rapidity of viral culture conversion following SARS-CoV-2 infection: a prospective cohort study

**DOI:** 10.1093/ofid/ofac492.088

**Published:** 2022-12-15

**Authors:** Madison Gilbert, Atarere Joseph, Jacquelyn Turcinovic, Scott Seitz, Cole Sher-Jan, Laura F White, Zhenwei Zhou, Mohammad Hossain, Devin Zebelean, Victoria Overbeck, Lynn Doucette-Stamm, Judy T Platt, Hannah E Nichols, Davidson H Hamer, Catherine M Klapperich, Karen R Jacobson, John Connor, Tara C Bouton

**Affiliations:** Boston Medical Center, Boston, Massachusetts; Boston Medical Center/ MedStar Health, Baltimore, MD; Boston University, Boston, Massachusetts; Boston University, Boston, Massachusetts; BUMC, Somerville, Massachusetts; Boston University School of Public Health, Boston, Massachusetts; Boston University, Boston, Massachusetts; Boston University, Boston, Massachusetts; Boston University School of Public Health, Boston, Massachusetts; Boston University School of Public Health, Boston, Massachusetts; Boston University, Boston, Massachusetts; Boston Iniversity, Boston, Massachusetts; Boston University, Boston, Massachusetts; Boston University School of Public Health, Boston, Massachusetts; Boston University College of Engineering, Boston, Massachusetts; Boston University School of Medicine, Boston, Massachusetts; Boston University, Boston, Massachusetts; Boston Medical Center and Boston University School of Medicine, Boston, Massachusetts

## Abstract

**Background:**

Vaccination is a fundamental element of pandemic control; however, insufficient data exists on vaccine’s impact on SARS-CoV-2 viral dynamics. We aimed to evaluate the relationship between time to negative viral culture conversion after diagnosis and time since most recent COVID-19 vaccination.

Scatterplot illustrating relationship between time from last dose of a COVID-19 vaccine and time to culture conversion. Both participants who only received their initial series of COVID-19 vaccination and those who received a COVID-19 vaccine booster dose were included. The black solid line shows the best fit for the Spearman correlation model; Gray shading denotes 95% confidence interval around this estimate. Spearman correlation coefficient, R, and p-value were estimates for the model.

Scatterplot illustrating relationship between time since completion of initial COVID-19 vaccine series and time to culture conversion among un-boosted participants. The vaccine type received has also been designated by plot labels. The black solid line shows the best fit for the Spearman correlation model; gray shading denotes 95% confidence interval around this estimate. Spearman correlation coefficient, R, and p-value were estimates for the model.

**Methods:**

CoViD Post-vax is longitudinal cohort study collecting baseline clinical questionnaires, and daily anterior nasal swabs and symptom screens on enrolled Boston University SARS-CoV-2 PCR-positive cases detected through a serial screening testing program or symptomatic testing. Data was collected from November 2021 to March 2022. Participants were excluded from analysis if they lacked at least one positive culture or did not culture convert during their study involvement. Scatter plots comparing time to culture conversion to time from initial vaccine series were created. We calculated spearman correlation coefficients to determine the relationship between time to culture conversion and time from last vaccination for all participants, those who completed the initial vaccine series (unboosted), and those who were boosted.

Scatterplot illustrating relationship between time since receiving a booster COVID-19 vaccine dose and time to culture conversion among boosted participants. The vaccine type received has also been designated by plot labels. The black solid line shows the best fit for the Spearman correlation model; Gray shading denotes 95% confidence interval around this estimate. Spearman correlation coefficient, R, and p-value were estimates for the model.

**Results:**

Of 54 CoViD Post-Vax participants included in this analysis, the mean age was 21 years (SD=2) and culture conversion occurred after a median of 4 days (IQR=3–5.75). There was no association between time to culture conversion and time since last dose of a COVID-19 vaccination (R= -0.13, *p*= .34). When stratified by vaccination status, there was no association between time to culture conversion and time since initial COVID-19 vaccine series (R= -0.25, *p*= .21, n=26) or time since COVID-19 booster vaccination (R= -0.24, *p*= .22, n=28).

**Conclusion:**

Our results show no significant relationship between time to culture conversion and time since most recent dose of COVID-19 vaccination in an initially culture positive, young, University-based cohort. More work needs to be done to understand the impact of symptom severity, disease burden, SARS-CoV-2 variants, and COVID-19 vaccine status on duration of transmissible SARS-CoV-2 infection.

**Disclosures:**

**Catherine M. Klapperich, Ph.D.**, BioSens8, LLC: Ownership Interest.

**Figure 1:**
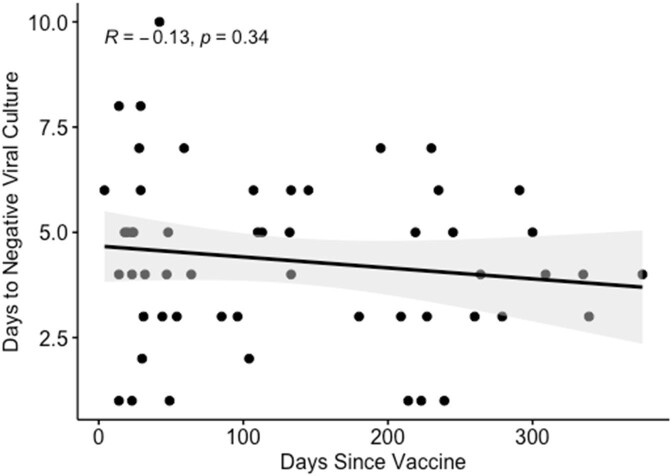
Scatterplot of time since COVID-19 vaccination and time to culture conversion (N=54).

**Figure 2:**
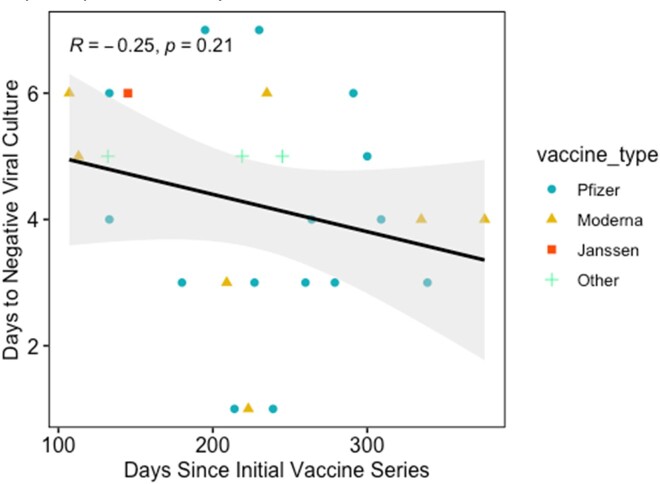
Scatterplot of time since initial COVID-19 vaccine series and time to culture conversion for participants who only received their initial series of a COVID-19 vaccine (not boosted, N=26).

**Figure 3:**
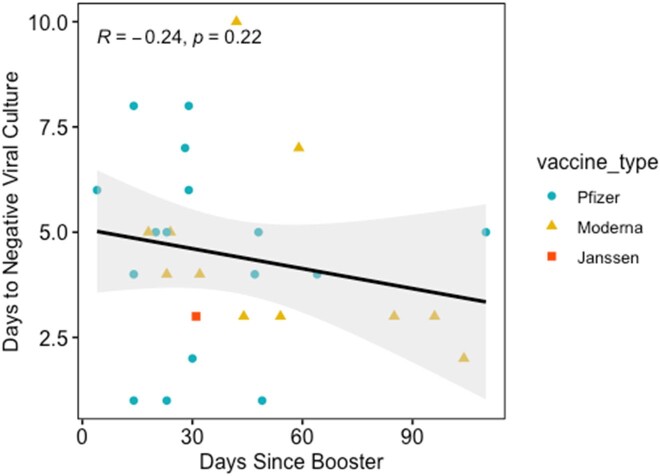
Scatterplot of time since booster of a COVID-19 vaccination and time to culture conversion for participants who received initial series of a COVID-19 vaccinations and a COVID-19 booster vaccination (N=28).

